# SOCS3, a Major Regulator of Infection and Inflammation

**DOI:** 10.3389/fimmu.2014.00058

**Published:** 2014-02-19

**Authors:** Berit Carow, Martin E. Rottenberg

**Affiliations:** ^1^Department of Microbiology, Tumor and Cell Biology, Karolinska Institutet, Stockholm, Sweden

**Keywords:** SOCS, JAK, STAT, STAT3, cytokine, IL-6, infection, autoimmunity

## Abstract

In this review, we describe the role of suppressor of cytokine signaling-3 (SOCS3) in modulating the outcome of infections and autoimmune diseases as well as the underlying mechanisms. SOCS3 regulates cytokine or hormone signaling usually preventing, but in some cases aggravating, a variety of diseases. A main role of SOCS3 results from its binding to both the JAK kinase and the cytokine receptor, which results in the inhibition of STAT3 activation. Available data also indicate that SOCS3 can regulate signaling via other STATs than STAT3 and also controls cellular pathways unrelated to STAT activation. SOCS3 might either act directly by hampering JAK activation or by mediating the ubiquitination and subsequent proteasome degradation of the cytokine/growth factor/hormone receptor. Inflammation and infection stimulate SOCS3 expression in different myeloid and lymphoid cell populations as well as in diverse non-hematopoietic cells. The accumulated data suggest a relevant program coordinated by SOCS3 in different cell populations, devoted to the control of immune homeostasis in physiological and pathological conditions such as infection and autoimmunity.

## Introduction

Cytokines are secreted proteins central for coordination of the initiation, maintenance, and termination of all types of immune responses, including host responses to infection, inflammation, and trauma. Most cytokines have a short half-life and are released locally at high concentrations. They interact with cell surface receptors triggering responses that include cell survival, activation, coordination of microbicidal effector mechanisms, and proliferation and differentiation depending on the type of cytokine and the nature of the target cell. Cytokines are released in response to environmental clues to ultimately preserve physiological homeostasis. Some of them are pro-inflammatory, and initiate an inflammatory response necessary to fight infection. Other cytokines are anti-inflammatory and serve to reduce inflammation and promote healing once the injury/infection/foreign body has been destroyed.

A tight control of cytokine release and of responses to cytokines is required for the defense against infections, the prevention of infection-associated immunopathology, and the correct development of immune cell populations. A number of different cellular and molecular mechanisms control the magnitude and duration of innate and adaptive immune responses and several of these mechanisms regulate cytokine responses.

Several cytokines, growth factors, and hormones utilize the Janus kinase–signal transducer and activator of transcription (JAK–STAT) pathway to transmit their information into the cell nucleus. In short, the cytokine receptor is activated after binding the cytokine. Binding to the cytokine activates the kinase function of JAK, a tyrosine kinase that binds to the receptor, which auto-phosphorylates itself, cross-phosphorylates a JAK molecule bound to the accompanying heterodimer chain of the cytokine receptor and also different tyrosine sites on the cytokine receptor (1). The STATs will then bind to the phosphorylated receptor though its SH2 domain and be phosphorylated by JAK. The phosphorylated STAT will undergo a conformational change, detach from the receptor, and then bind to another phosphorylated STAT. STAT homo- or hetero-dimers translocate into the cell nucleus, bind to target genes, and promote their transcription (2). It is fascinating that only four JAK and seven STAT molecules mediate a huge diversity of biological effects, in face of their highly specific functions in the control of various immune responses revealed by genetic knockout studies (3). Such specificity is due to their individual patterns of activation by particular cytokine receptors and to some extent by their individual DNA sequence recognition preferences (4).

Janus kinase–signal transducer and activator of transcription pathways are tightly regulated at many steps through distinct mechanisms, including phosphotyrosine phosphatases (PTPs), protein inhibitor of activated STAT (PIAS), and suppressor of cytokine signaling (SOCS) proteins (5).

Phosphotyrosine phosphatases participate in the regulation of the JAK/STAT signaling pathway and have important implications in physiology and diseases (6).

The PIAS regulate the activity of many transcription factors, including STATs (7). Different PIAS bind different STATs and probably act by inhibiting their DNA binding or by recruiting histone deacetylases (8). Neither PTPs nor PIAS exclusively inhibit the JAK/STAT pathways, but are also main regulators of other cellular functions.

Suppressor of cytokine signaling is a protein family of eight members (SOCS1–7 and CIS) that inhibit STAT activation by many, but not all, JAK–STAT activating receptors. Experiments in different genetically manipulated mice have demonstrated a crucial role of SOCS molecules in pathophysiology. For example, SOCS1-deficient mice die within 3 weeks of birth due to severe systemic inflammation resulting from uncontrolled interferon-γ (IFN-γ) signaling (9). SOCS2-deficient mice develop gigantism due to enhanced responses to growth hormone (10). Mice lacking SOCS3, the objective of this review, die perinatally probably due to defective placental formation (11, 12).

Non-canonical ways of STAT activation have been shown (13). For example, unphosphorylated forms of STAT might function as transcription factors, modifiers of transcription factors, regulate the heterochromatin formation, or even possess non-transcriptional and extra-nuclear functions (14–16). The epidermal growth factor receptor (EGFR) catalyzes the tyrosine phosphorylation of STAT3 in response to EGF (17), and the intrinsic kinase activity of the receptor, but not of any JAK, is required for this reaction (18). Of importance for this review, these non-canonical STAT activation pathways might be independent of SOCS control (19).

Suppressor of cytokine signaling-3 regulates STAT3 activation in response to cytokines using the gp130 receptor. gp130 (CD130) forms part of the receptor complex for cytokines belonging to the IL-6 family, including IL-6, IL-11, IL-27, leukemia inhibitory factor (LIF), oncostatin M (OSM), ciliary neurotrophic factor (CNTF), cardiotrophin-1, and cardiotrophin-like cytokine. The functions of these cytokines encompass both unique but also partially redundant activities on multiple cell lineages (20). Gp130 is expressed in almost all organs and targeted deletion of the *gp130* gene in mice results in embryonic lethality (21). SOCS3 also regulates the response to cytokines, growth factors, and hormones that are independent of gp130, such as the IL-12R, granulocyte-colony stimulation factor (G-CSF), leptin, insulin, and others, usually inhibiting STAT3 activation (22), but also other receptors that do not activate STAT3 (Table 1).

**Table 1:** **Molecules regulated by SOCS3**.

		Receptor	Cytokine or pathway dysregulated	Reference
STAT activators	STAT3		gp130	(23–25)
			IL-6	(23, 24)
			IL-11	(26)
			IL-27	(27)
			OSM	(28)
			CT-1	(29)
			LIF	(30, 31)
		Non-gp130 receptors		
		G-CSF	G-CSF R	(32)
		IL-23R	IL-23	(33)
		EPO-R	EPO	(34)
		Leptin-R	Leptin	(35, 36)
	STAT4	IL-12 Rb2	IL-12	(37)
	STAT1	Gp130	IL-6	(30)

STAT-independent molecules			Indoleamine dioxygenase	(38)
		CD33-family	CD33	(39)
	NF-κB		Siglec	(40)
			TRAF6	(41)
			iκB	(42)
	Others		Pyruvate kinase M2	(43)
		IRS-1	Insulin	(44)
			IRS-2	Insulin	(44)

Studies in different mouse models have proven the critical importance of SOCS3 in restraining inflammation and allowing optimal levels of protective immune responses against infections. We here review the latest advances in SOCS3 biology, focusing on its role in the control of infection and inflammation.

## SOCS Structure and Function

In 1995, Yoshimura et al. identified cytokine-induced STAT inhibitor (CIS), the first member of the SOCS family (45). A couple of years later, SOCS1 was shown to inhibit STAT signaling (46, 47), and the presence of several SOCS proteins with homologous conformations were predicted. Eight of these anticipated molecules in the human genome were subsequently cloned (SOCS1–7, CIS) (48–50).

All SOCS proteins have a central SH2 domain and a short C-terminal domain, the SOCS box as well as an N-terminal domain of varying length. SOCS inhibits the receptor complex by ubiquitination and subsequent proteasome-mediated degradation. SOCS proteins act as substrate adapters: the SOCS box associates with a complex containing elongin B and C and this complex then binds Cullin-5 (51, 52). Since SOCS proteins contain a central SH2 domain, any tyrosine phosphorylated signaling intermediate (phospho-JAK, phospho-STAT, phosphorylated receptors) is a conceivable substrate. Thus, the SH2 domain functions as an adapter bringing ubiquitin ligases close to kinase-activated signaling proteins, mediating their degradation (52).

However, SOCS1 and 3, the most studied molecules of the family, are partially active in absence of their SOCS box domain (53). Moreover, the SOCS box of SOCS1 and SOCS3 binds with lower affinity to the E3 ubiquitin ligase than those of SOCS2, 4–7, and CIS (52).

Instead, SOCS3 and SOCS1, but not the other members of the SOCSs family, bind the JAKs directly inhibiting their kinase activity. Studies using truncated or chimeric forms of SOCS proteins showed that SOCS1 and SOCS3 contained a short N-terminal kinase inhibitory region (KIR) resembling a JAK substrate, which allows them to suppress signaling by direct inhibition of JAK’s catalytic activity (54, 55).

There are four mammalian JAKs (JAK1–3 and TYK2). SOCS3 has been shown to inhibit JAK1, JAK2, and TYK2 but not JAK3 (56). Despite the ability of SOCS3 to bind to and inhibit JAKs, deletion of individual SOCS genes in mice has revealed an exquisite specificity for particular cytokine receptor combinations rather than specific JAKs. This specificity is provided by the binding of the SH2 domain of the SOCS proteins to the gp130 cytokine receptor (57).

**Figure 1:**
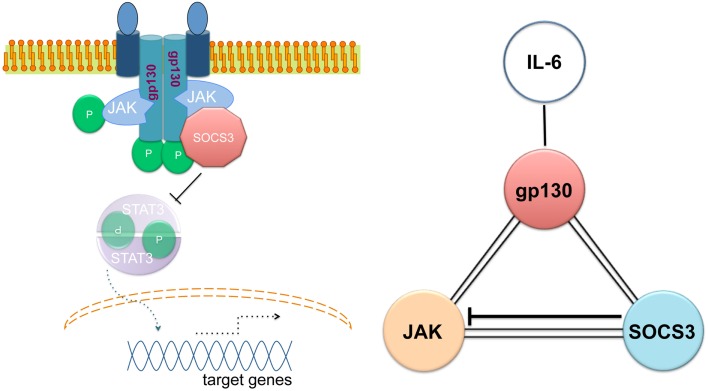
**Simultaneous binding of SOCS3 to JAK and the gp130 cytokine receptor**. Adapted from Ref. (56).

The ability of SOCS3 to simultaneously bind to JAK and to the cytokine receptor explains the specificity of the suppression. SOCS3 generates a ternary complex in which each moiety is directly bound to the other two with an overall affinity higher than the individual associations (Figure 1) (58). In other words, SOCS3 inhibits JAK’s enzymatic activity by blocking substrate binding and gains specificity of action by only binding tightly to JAK when the kinase is attached to specific receptors. SOCS3 binds JAK and gp130 receptor simultaneously, using two opposing surfaces: while the phosphotyrosine-binding groove on the SOCS3 SH2 domain is occupied by the gp130 receptor, a subdomain in the SH2 domain of SOCS3 is also required for inhibition of JAK, binding in a phospho-independent manner to a non-canonical surface of JAK2 (58, 59). The KIR of SOCS3 occludes the substrate-binding groove on JAK2.

On the other hand, a relevant role for the SOCS box in the SOCS3-mediated proteasomal degradation resulting in the regulation of cytokine signaling through some receptors (i.e., the G-CSF receptor) has been shown (60, 61). Thereby, SOCS3 regulates a STAT3-mediated chemokine and chemokine receptor function within the bone marrow, and plays a central role in the neutrophil mobilization response (62).

The SOCS3 structure does not exclude an apparent specificity of SOCS3 as an inhibitor of the activation of other STATs than STAT3. As indicated below, SOCS3 also inhibits IL-6-mediated STAT1 and STAT4 activation (30, 63).

## SOCS3 and STAT3

Loss of SOCS3 *in vivo* has profound effects on placental development, inflammation, fat-induced weight gain, and insulin sensitivity.

Genetic deletion of SOCS3 leads to mid-gestational embryonic lethality due to increased STAT3 and MAP kinase activation (11, 12). Lack of suppression of LIF and fetal erythropoiesis signaling had been shown to account for the lethality of *Socs3*^−/−^ mice (11, 12). LIF belongs to the IL-6 family and is involved in blastocyst implantation. LIFR deficit rescued the *Socs3*^−/−^ placental defect and embryonic lethality. These double KO mice died by 190 days of age due to neutrophilia accompanied by neutrophil tissular infiltration (64). However, mice with a mutation in the gp130 chain of the IL-6 receptor family that impairs binding of SOCS3 (*gp130^F/F^* mice) display no early lethality (65), indicating that altered LIFR signaling is not the only cause of the mid-gestational death of *Socs3*^−/−^ mice (Table 2).

**Table 2:** **Mouse models to study SOCS3 function**.

Genotype	Population targeted	Cytokine involved	Reference
*Socs3*^−/−^	All	LIF, EPO	(11, 12, 64)
*Socs3*^±^	All	Leptin	(66, 67)
Gp130^F/F^*	All	Gp130	(26, 27, 65, 68)
*Gp130^F/F^ Il-6*^−/−^	All	Non IL-6, gp130 mediated cytokines	(69, 70)
*Gp130^F/F^ Stat3*^±^	All	Role of SHP2 in inhibition via gp130	(26, 69, 71)
*Gp130^fl/fl^ LysM cre*	Myeloid cells		(72)
*Socs3^fl/fl^ Lck cre/CD4 cre*	T cells	IL-23, IL-12	(27, 73 –79)
*Socs3^fl/fl^ LysM cre*	Myeloid cells	IL-6, G-CSF, IL-27	(63, 74, 80 –84)
*Socs3^fl/fl^ Nes cre*	Neural cells	Leptin	(85)
*Socs3^fl/fl^ Syn cre*	Neural cells	Leptin	(85)
*Socs3^fl/fl^ aP2 cre*	Adipose tissue	Insulin	(67)
*Socs3^fl/fl^ Alb cre*	Liver cells	Insulin	(63)
*Socs3^fl/fl^ MBP cre*	Oligodendrocytes	LIF	(86, 87)
*Socs3^fl/fl^ vav cre*	Hematopoietic and endothelial		(75, 88 –90)
*Socs3^fl/fl^ Tie cre*	Hematopoietic stem cells	G-CSF	(81)
*Socs3^fl/fl^ MMTV-LTR*	Glands, seminal vesicle, skin, and B and T cells	IL-23 (T cells *in vitro*)	(33)
*Socs3^fl/fl^ Mx cre*	Hematopoietic cells during type I IFN response		(79)
*Lck-SOCS3* Tg	T cells	Th2 cytokines	(91 –94)
*Socs3^fl/fl^ Adenovirus cre*	Liver cells		(95)
SOCS3 adenovirus	Local injection	IL-6, TNF, IL-1β	(96)
Cell-penetrating SOCS3	All		(97)

Mice with a deletion of SOCS3 in hematopoietic cells (*Socs3^fl/fl^* *vav cre*) have been shown to develop a severe inflammatory disease during adult life (88). IL-6 was not critical in regulating the severity of this spontaneous inflammatory disease but played a role in the onset (89). Since *Socs3*^−/−^ but not *Socs3^fl/fl^* *vav cre* mice die during gestation, SOCS3 probably impairs lethal cytokine or growth factor responses in non-hematopoietic cells.

*Gp130^F/F^* mice spontaneously develop lymphadenopathy, splenomegaly, and gastric hyperplasia (70). The basis for this phenotype is complex, but it appears that the enhanced ability of IL-11, rather than IL-6, to activate STAT3 and STAT1 in absence of SOCS3 promotes inflammation and cancer (26, 69).

On the other hand, enhanced IL-6 responses accounted for the enhanced susceptibility of *Socs3^fl/fl^* *vav cre* or *Socs3^fl/fl^* *LysM cre* (deficient in SOCS3 in myeloid cells) mice to induced inflammatory diseases like rheumatoid arthritis (RA) or experimental autoimmune encephalomyelitis (EAE) (90, 98). *Gp130^F/F^* mice spontaneously develop a RA-like disease that is accelerated by IL-6 administration (99). Accordingly, adenoviral-delivered SOCS3 reduced joint inflammation in mice with arthritis via inhibition of IL-6 signaling (96).

Suppressor of cytokine signaling-3 is not an essential regulator of IL-10 or IFN-γ responses (80). In the absence of SOCS3 in hematopoietic or myeloid cells, IL-6 acts like IL-10 and attenuates macrophage secretion of TNF and IL-12 after LPS stimulation (80). Furthermore, *Socs3^fl/fl^ LysM cre* mice were protected from the lethal effects of galactosamine and LPS administration, a model that is dependent on TNF-induced liver failure (100). These results were somewhat contrary to the expected: if IL-6 is a pro-inflammatory cytokine, removal of its inhibitor should result in more, rather than in less, inflammation. Paradoxically, the pro-inflammatory IL-6 and the anti-inflammatory IL-10, generating nearly opposing cellular responses, both activate STAT3 after binding to their receptors. The kinetic of STAT3 activation was pointed as the putative cause of SOCS3’s effect: the suppressive effect of IL-6 signaling on TNF and IL-12 secretion in absence of SOCS3 was explained to be due to a sustained STAT3 activation (101, 102). In line with this, a transient activation of the IL-10 receptor elicited an IL-6-like response (102). However, how the duration of STAT3 activation can direct distinct responses is far from being understood.

Interestingly, after the initial phosphorylation of STAT3 in response to IL-6 followed by a subsequent inhibition by SOCS3, a second wave of activation leads to the re-phosphorylation of STAT3 (101). It has been recently shown that re-phosphorylation requires an IL-6-dependent association of IL-6R and EGFR without involvement of gp130. STAT3 phosphorylation thus might continue to be driven for many hours by this two-receptor complex that is immune to inhibition by SOCS3 (19).

The anti-inflammatory responses of SOCS3-deficient macrophages or dendritic cells (DCs) are not restricted to diminished TNF- or IL-12 levels but also have been shown to increase the secretion of IL-10, expand the numbers of regulatory T (Treg) cells, and decrease MHC-II expression levels (81). In line with this, SOCS3 inhibited the TGFβ1/Smad3 signaling pathway, leading to enhanced LPS responses in macrophages (103). In contrast, SOCS3 expression in myeloid cells has been shown to mediate LPS-induced lung injury (82).

Deletion of SOCS3 in hematopoietic cells surprisingly also enhanced the expression of STAT1-stimulated genes in response to IL-6 (30, 63). The activation of STAT1 in SOCS3-deficient cells is probably due to a more dramatic inhibition of STAT1 than of STAT3 by SOCS3. A differential effect of SOCS3 on STAT3 and STAT1 has lately been used to explain the preferential development of either M1 (classically activated) (83, 98) or M2 (alternatively activated) macrophages from *Socs3^fl/fl^* *Lys M cre* mice (104, 105). M1 macrophages are differentiated after IFN-γ-stimulation, while IL-4 and/or IL-10 activate an alternative M2 program. Importantly, in these contradictory studies on the role of SOCS in the regulation of M1 and M2 polarization, *Socs3^fl/fl^ LysM cre* mice showed either increased resistance or susceptibility to LPS-induced septic shock (83, 104). Altogether, it is still unknown whether SOCS3 determines whether a cellular response to IL-6 is pro- or anti-inflammatory. The fine regulation exerted by SOCS3 needs further understanding.

Mechanisms regulating macrophage polarization via SOCS3 were also studied. The Notch signaling pathway specifies cell differentiation during development (106). Activation of Notch signaling increased M1 macrophage differentiation, no matter whether M1 or M2 inducers were applied. When Notch signaling was blocked, even the M1 inducers induced a M2 response. Interestingly, Notch signaling has been shown to regulate macrophage polarization via SOCS3 (107): in the presence of an inhibitor of Notch signaling, macrophages over-expressing SOCS3 showed restored M1 polarization in response to LPS (107). Notch is also involved in SOCS3 up-regulation following a mycobacterial infection (108).

Perturbed hematopoiesis was observed in mice lacking SOCS3 in myeloid cells (88, 109). IL-6 played a role in the onset of this severe disease (89). These mutant mice were hyper-responsive to injected G-CSF, showing exaggerated neutrophilia, mobilization of progenitor cells into the blood, splenomegaly, and an accelerated disease (88, 110). SOCS3 expression was stimulated by G-CSF and SOCS3 directly bound to a phosphotyrosine on the G-CSF-receptor (32). The effect of SOCS3 in the regulation of neutrophil biology might well underlie its protective activity in development of spontaneous or induced inflammatory diseases.

## SOCS3 but Not STAT3

Besides inhibiting JAK–STAT-mediated signals, SOCS3 has been suggested to hamper other signaling pathways (Table 1). SOCS3 has been shown to bind to indoleamine dioxygenase (IDO), targeting the complex for ubiquitination in DCs. Thus, acting at a post-transcriptional level it antagonizes IDO-dependent tolerogenic signals in DCs and converts them into immunogenic (38, 111).

Suppressor of cytokine signaling-3 has also been shown to bind and degrade CD33 or Siglec 3, blocking CD33-mediated inhibition of proliferation (39). Siglec 7, another CD33-family receptor, has also been found to be bound and degraded by SOCS3 (40).

It has also been proposed that SOCS3 can bind to the insulin receptor (IR) or the insulin receptor substrate-1 (IRS-1) targeting them for proteasomal degradation and regulating thereby insulin sensitivity (44, 112–114).

Suppressor of cytokine signaling-3 has also been shown to directly interact with SMAD3 inhibiting the responses to TGF-β (103). On the other hand, TGF-β has been shown to induce the expression of SOCS3, allowing TNF-induced osteoclast formation (115).

Different microorganisms including mycobacteria stimulate the expression of SOCS3. PPE18, associated with mycobacterial virulence has been shown to increase SOCS3 expression and its tyrosine phosphorylation. Surprisingly phospho-SOCS3 was found to bind to iκB hampering its degradation and thereby blocking NF-κB activation (42). Another study indicated that SOCS3 inhibits both the IL-1-induced NF-κB and JNK/p38 pathways by binding the upstream molecule TRAF6 and preventing its function (41). Similarly, IL-25, a member of the IL-17 cytokine family that promotes Th2 responses, has been shown to hamper the LPS-induced, MAP kinase p38-dependent secretion of pro-inflammatory cytokines in a SOCS3-mediated manner (116).

The functional role of a JAK/STAT-independent SOCS3 regulation of these molecules remains to be validated. However, these results suggest that the characterization of SOCS3 as a STAT3 inhibitor is oversimplified.

## SOCS3 and T Cells

The role of SOCS3 in T cell development has been somewhat overlooked. SOCS3 is expressed in the double negative (early) stage of thymocyte differentiation (73), a stage at which T cells determine their expression of γδ+ or αβ+ T cell receptors (TCR). Most T cells have a TCR composed of two chains called α and β (so called αβ+ T cells). In contrast, a small subset of T cells has a TCR made up by a γ and a δ chain. These γδ+ T cells are more of an innate T cell subset, with a relatively invariant TCR and lower if any requirement of antigen recognition or processing for activation. Recent experiments from our laboratory showed that the thymus, or secondary immune organs of either neonatal or adult mice with T cells lacking SOCS3 have an increased frequency of γδ+ T cells compared to controls (74). Thus, SOCS3 regulates T cell development in the thymus. However, the precise mechanisms utilized by SOCS3 remain unexplored.

IL-27 has an anti-inflammatory role during immune responses, such as CD28-mediated IL-2 secretion. SOCS3-deficient CD8+ T cells showed higher proliferation in response to TCR ligation than wild-type cells despite a normal activation of signaling pathways downstream the TCR and CD28 receptors. Suppression of IL-27 signaling was found to substantially reduce the increased anti-CD3-induced proliferation of SOCS3-deficient T cells (27). Thus, SOCS3 mediates the anti-proliferative role of IL-27. The expression of SOCS3 is induced by IL-27 in mouse and human cells (117), and mediates the inhibitory effect of IL-27 (118). In line with this, SOCS3 deficiency in donor T cells promoted acute GVHD mortality (75).

Cytokines can direct CD4+ Th0 cells into Th1, Th2, Th17, or Treg cell lineages. Th2 cells contain higher amounts of SOCS3 compared to Th1 cells (119). SOCS3 has been also suggested to inhibit IL-12-induced STAT4 activation by direct binding to IL-12Rβ2 (37), the IL-12R subunit that is not shared with the IL-23R.

In line with this, the increased SOCS3 expression in T cells correlated with the severity of asthma and atopic dermatitis or with the Th1-mediated condition psoriasis (91, 120, 121). Furthermore, defined haplotypes of SOCS3 have been linked with atopic dermatitis in childhood cohorts (121).

Accordingly, over-expression of SOCS3 in T cells inhibits Th1 and promotes Th2 development suggesting that SOCS3 stimulates allergic responses (91). T cell-specific expression of SOCS3 also aggravated allergic conjunctivitis, a Th2-mediated model of disease (92). Inhibition of SOCS3 expression in T cells exhibited markedly suppressed airway hyper-responsiveness and eosinophilia (76, 77). Mice with T cells over-expressing SOCS3 also showed a delayed onset of EAE and restricted Th17 differentiation (122).

*In vitro*, SOCS3-deficient CD4+ T cells produced more TGF-β and IL-10 but less IL-4 than control T cells (77). TGF-β inhibits IL-6- and IL-21-induced SOCS3 expression, thus enhancing as well as prolonging STAT3 activation in naive T cells (123). Thus, TGF-β production is inhibited by SOCS3 and vice versa.

Th17 cell differentiation is induced by IL-6 and IL-21 in the presence of TGF-β through the activation of STAT3 (124). STAT3 induces the orphan nuclear receptor RORγt, which directs Th17 cell differentiation by producing the IL-23 receptor (124). The survival and expansion of committed murine Th17 cells requires IL-23 (125). The critical role of STAT3 in Th17 differentiation was also confirmed in human patients lacking functional STAT3 (126). SOCS3 was found to be a major negative regulator of IL-23-mediated STAT3 phosphorylation and Th17 generation (33, 78, 123).

Suppressor of cytokine signaling-3 expression in fibroblasts has been shown to participate in Th17 development. IL-17 increased a STAT3-dependent IL-6 expression in fibroblasts. IL-6 secretion was enhanced in mice deficient for SOCS3 in fibroblasts resulting in enhanced Th17 levels (99). Thus, SOCS3-mediated regulation of cytokine responses in T and non-T cell lineages impairs Th17 differentiation.

IL-17 has been also shown to increase collagen fiber formation and fibrous cap development in atherosclerosis models (127). The strength of the cap given by the collagen fibers prevents plaque rupture, a condition that elicits thrombosis and infarction in patients. The increased Th17 frequency among SOCS3-deficient T cells reduced atherosclerotic lesion development, which was dependent on IL-17. Accordingly, the over-expression of SOCS3 in T cells reduced IL-17 levels and accelerated atherosclerosis and severe aortic aneurysm formation (78, 93).

Transplanted neural progenitor cells (NPCs) differentiate into neural cells and provide the means to repair demyelinated nerve fibers in the CNS in neuroinflammatory diseases like multiple sclerosis. Surprisingly, NPC treatment has been shown to suppress self-reactive T cells and control tissue inflammation via the secretion of LIF. LIF inhibits Th17 differentiation by inducing SOCS3 (128). The LIF receptor is expressed on T cells. Thus, two members of the IL-6 family, IL-6 and LIF, show opposing effects on Th17 development: while IL-6 is required for Th17 development, LIF counteracts it. During Th17 development, IL-6 activates while LIF inhibits STAT3 activation. In line, inhibition of SOCS3 with specific siRNA hampered the inhibitory effects of LIF on Th17 development (128).

As stated above, SOCS3 expression in non-T cells can direct T cell differentiation and function. DCs are required for T cell selection, differentiation, and activation. SOCS3-deficient DCs expressed lower levels of MHC II, CD40, CD86, and IL-12 (81, 111). As discussed, SOCS3 expression in DCs antagonized a tolerogenic CTLA-4 activity by direct interaction and degradation of IDO (38).

Contrary to these observations, DCs transduced with SOCS3 significantly inhibited IL-12 and IL-23 activation of STAT4 and STAT3, respectively. Together with an inhibition of MHC-II and CD86 expression, SOCS3 promoted a Th2 differentiation that hampered EAE development (129). SOCS3 might also interact with pyruvate kinase-M2 to decrease ATP production, accounting for such a DC dysfunction (43).

## SOCS3 and Infections

Suppressor of cytokine signaling-3 expression is stimulated by cytokine or innate immune receptor agonists present in viruses, bacteria, and parasites (22, 130). Due to the multiple binding partners of SOCS3, it is hard to predict its role in different infections. Of importance, SOCS3 expression is induced by several cytokines to which the receptor has no binding site (i.e., IL-10), probably to inhibit trans-signaling via other cytokines (131). Table 3 provides a summary of the role of SOCS3 in several infections.

**Table 3:** **Role of SOCS3 in infections**.

Pathogen	Mechanism	Pathology	Pathogen control	Reference
HSV-1	↓IFN-αβ signaling		Worsened	(132)
RSV	↓IFN-αβ signaling		Worsened	(133)
SIV	↓Th17 responses		Worsened	(134, 135)
	↓IFN-αβ signaling			
HCV/HIV-1	↓IFN-αβ signaling		↓Response to IFN-therapy	(136)
EBV	↓IFN-αβ secretion and signaling			(137)
HBV	↓Hepatic insulin signaling			(138)
HCV	↓Insulin signaling			(139)
HCV			↓Response to IFN-therapy	(140–142)
Influenza A virus	↓IRF3 and NF-κB		Worsened	(143, 144)
	↓IFN-αβ signaling			
LCMV	↓T cell activation and memory	↑If SOCS3 is deleted in all cells	Worsened	(79, 89)
		↓If SOCS3 is deleted in T cells	None	
LCMV	↑T cell memory			(145)
*L. major*	↓TGF-β/IL-10 production by T cells (T cell knockdown)		Improved	(77)
*L. major*	↑IL-4 (T cell transgene)	Worsened	Worsened	(94)
*T. gondii*	↑IL-12 induction in dendritic cells	Improved	Improved	(68, 84)
*M. tuberculosis*	IL-12 induction by DCs	Improved	Improved	(74, 146)
	γδ+ T cells formation	Improved	Improved	

### Viral infections

The lymphocytic choriomeningitis virus (LCMV) clone 13 triggers insufficient CD8+ T cell responses and thus persists indefinitely in the host. LCMV promotes high expression of SOCS3 in T cells, resulting in impaired antiviral functions and viral persistence. Treatment of LCMV-infected mice with IL-7 repressed SOCS3 expression and enhanced T cell effector functions and viral clearance. Mechanistically, a reduction of SOCS3 allowed the differentiation of Th17 cells. IL-17 stimulates the expression of IL-6 that mediates survival and function of antiviral T cells. IL-7 also promotes IL-22 secretion, which protects against immune tissue destruction (79). On the other hand, the deletion of SOCS3 in all hematopoietic cells induced an IL-6 dependent early lethality in LCMV-infected mice despite the viral clearance (89).

Hepatitis C virus (HCV) infection is a major cause of chronic liver disease, affecting 170 million persons worldwide. The most effective current treatment for chronic HCV is the combination of type I IFN and ribavirin, a nucleoside analog. Hepatic SOCS3 expression is associated with non-response to therapy in human HCV patients (136, 140). Particular SOCS3 (-4874 AA) genotypes express SOCS3 at elevated levels and consequently have a poorer response to therapy (140–142). One microRNA (miR122) modulated the response to type I IFNs: silencing of miR122 enhanced IFN-induced ISRE activity by decreasing the expression of SOCS3. Interestingly, such decrease in SOCS3 levels was not mediated by microRNA target gene suppression, but rather by enhanced methylation at the SOCS3 gene promoter (147).

Alike, the infection of human cells by either Epstein–Barr virus or Herpes simplex virus (HSV) has been shown to stimulate SOCS3 expression that suppresses type I IFN production and responses (132, 137). Silencing of SOCS3 using anti-sense nucleotides significantly hampered replication of HSV-1 (132).

In studies of simian immunodeficiency virus (SIV) infection, a significant increase in markers of microbial translocation that correlate with suppressed Th17 responses was found. Elevated expression of SOCS3 could potentially play a role in suppressing IL-17 expression during an acute SIV infection (134).

In a SIV/macaque model of HIV-associated dementia, SOCS3 expression correlated with recurrence of viral replication and onset of CNS disease. SOCS3 expression attenuated the response of macrophages to IFN-β. Thus, SOCS3 may allow HIV-1 to evade the protective innate immune response in the CNS by overcoming the inhibitory effect of IFN-β on HIV-1 replication within the macrophages (135).

### Bacterial and parasitic infections

The role of SOCS3 in the outcome of infection with intracellular bacteria and parasite has also been studied.

*Toxoplasma gondii* is an intracellular eukaryotic pathogen that causes toxoplasmosis, a life-threatening condition, which includes congenital disease and infection in immunocompromised individuals. Toxoplasma possesses a secretory organelle called rhoptry. Infection with *T. gondii* diminished innate immune responses due to the inoculation of the rhoptry ROP16 kinase (148). ROP16 activated STAT3, which stimulated SOCS3 expression that in turn diminished STAT3 activation (84). *Socs3^fl/fl^ LysM cre* or *gp130^F/F^* mice succumbed to *T. gondii* infection, and resistance could be restored by neutralization of IL-6 (68, 84). Diminished IL-12 secretion by SOCS3-deficient DCs was suggested to impair IFN-γ secretion by antigen-specific T cells, probably contributing to the increased susceptibility to *T. gondii* infection of the mutant mice (68).

*Mycobacterium tuberculosis* causes the highest mortality to a single pathogen worldwide. Only 10% of infected individuals will manifest active tuberculosis while most apparently control the infection by an appropriate immune response. Infection with mycobacteria enhanced the expression of SOCS3 in phagocytes (149).

**Figure 2:**
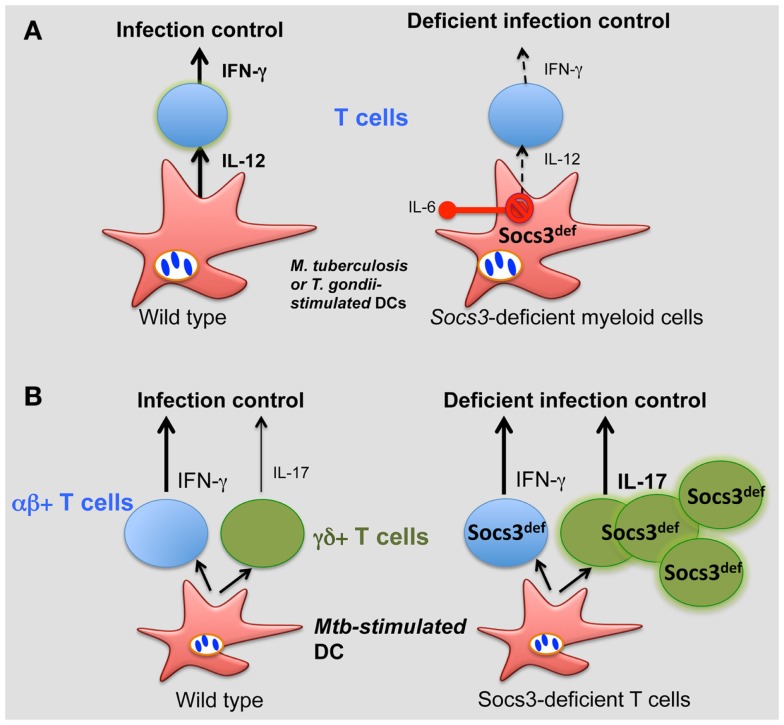
**Role of SOCS3 in the control of infection**. **(A)** During *M. tuberculosis* and *T. gondii* infection, SOCS3 is induced in macrophages and DCs prevents the IL-6-mediated inhibition of IL-12 secretion, promoting a CD4+ cell-dependent IFN-γ expression. **(B)** SOCS3 controls the development of γδ+ T cells. Such control is critical for proper protection against *M. tuberculosis*.

We demonstrated a critical role for SOCS3 expression by myeloid and lymphoid cells in resistance against *M. tuberculosis* (74). All *Socs3^fl/fl^ LysM cre, Socs3^fl/fl^ lck cre* (with SOCS3-deficient T cells), and *gp130^F/F^* mice showed increased susceptibility to infection with *M. tuberculosis*. SOCS3 binding to gp130 in myeloid cells conveyed resistance to *M. tuberculosis* infection via the regulation of IL-6/STAT3 signaling. SOCS3 was redundant for mycobacterial control by macrophages *in vitro*. Instead, SOCS3 expression in infected macrophages and DCs prevented the IL-6-mediated inhibition of IL-12 secretion and contributed to a timely CD4+ cell-dependent IFN-γ expression (Figure 2A).

Interestingly, γδ+ rather than αβ+ T cells accounted for the susceptibility to infection of *Socs3^fl/fl^ lck cre* mice. γδ+ T cells’ numbers in SOCS3-deficient mice were increased (independently of infection) and accounted for the exacerbated susceptibility to disease of these mutant mice (Figure 2B). Surprisingly, opposed to αβ γδ+ T cells have been shown to be redundant in protection of mice against *M. tuberculosis*. In line with this, severity of tuberculosis in humans was inversely associated with the expression of SOCS3 (146).

Macrophage/neutrophil-specific gp130-deficiency, the over-expression of soluble gp130 (sgp130) or the administration of sgp30 did no affect mycobacterial loads or pathology (72, 150). Similarly, *Il-6*^−/−^ mice have an unimpaired generation of protective memory responses and control of mycobacterial growth (151).

*Socs3^fl/fl^ lck cre* mice showed a worsened disease progression after infection with *Leishmania major*, which associated to the hyper-production of IL-10 and TGF-β (77). On the other hand, transgenic mice over-expressing the SOCS3 gene in T cells (*Lck-SOCS3* Tg mice) were also susceptible to infection by *L. major* due to an increased IL-4 secretion (94), altogether suggesting that a tight regulation of SOCS3 expression in T cells is crucial for disease control during infection by *L. major*.

Thus, SOCS3 seems to be detrimental for controlling viral infections by impairing proper type I IFN in viral defense. On the other hand, SOCS3 enables suitable IL-12 secretion by DCs for bacterial parasite control. However, a tight regulation of SOCS3 expression in T cells is critical for determining the outcome of infection.

## SOCS3 in Non-Hematopoietic Cells

Suppressor of cytokine signaling-3 plays important physiological roles in non-hematopoietic cells such as neurons, muscle cells, hepatocytes, fibroblasts, and adipocytes. For example, SOCS3 controls neurite outgrowth in dorsal root ganglia, insulin resistance in adipocytes, and is associated with age-related blunted muscle stem cell responses (152–154).

During inflammation, SOCS3 is expressed in epithelial and lamina propria cells in the colon of mice with intestinal bowel disease, in human ulcerative colitis and Crohn’s disease patients (155), and in synovial fibroblasts of RA patients (96). In human atherosclerotic lesions, vascular smooth muscle cells and macrophage expressed SOCS3 (156). The over-expression of SOCS3 in T cells reduced IL-17 and accelerated atherosclerosis whereas the *in vivo* treatment with anti-sense oligodeoxynucleotides targeting SOCS3 exacerbated the atherosclerotic process in *ApoE*^−/−^ mice (78).

These findings are consistent with the idea that IL-6-related cytokines promote while SOCS3 prevents chronic disease progression. However, no dogmas can be concluded since a pathogenetic involvement of SOCS3 has also been shown: in obesity, chronic JAK–STAT3 activation in the CNS by increased circulating leptin levels lead to the development of leptin resistance, whereas in the peripheral organs chronic IL-6-induced STAT3 activation impairs insulin action (157). Leptin is secreted from adipocytes proportionally to the amount of fat stored in the white adipose tissue and acts on a group of neurons of the hypothalamus to suppress food intake and to increase energy expenditure (158). In obesity, expansion of white adipose tissue increases leptin levels, but the protein does not convey its biological effects. SOCS3 expression in the CNS is largely increased in obesity. SOCS3 binds to the leptin receptor and thereby limits leptin action (35, 36, 159). Mice lacking SOCS3 in this particular population of neurons are protected from the development of diet-induced obesity and maintain central leptin sensitivity (66, 85, 160).

In agreement with these results, circulating levels of cytokines including IL-6 and TNF-α impair insulin signaling in peripheral organs. IL-6 increases SOCS3 levels in adipose tissues, muscle cells, and hepatocytes. Mice with deficient SOCS3 expression in adipose tissues were protected against the development of obesity-associated insulin resistance (67). As indicated above, SOCS3 impairs insulin action by binding to the insulin receptor or the IRS-1 and IRS-2 leading to their ubiquitination and degradation or by inhibition of receptor tyrosine phosphorylation (44, 113).

IL-6-related cytokines are induced by and play a protective role in the injured myocardium. Mice with a SOCS3 deletion in cardiomyocytes showed higher activation of STAT3, AKT, and ERK1/2 pathways, and reduced mitochondrial damage, oxidative stress, and inflammation resulting in the prevention of myocardial injury (161).

## Pharmacological Targeting of SOCS3

The multiple effects of SOCS3 in different cell lines and experimental models call for thorough investigations to clarify its main mechanisms and targets. Strategies increasing SOCS3 expression or mimicking its consequences (i.e., hampering STAT3 activation) might be appropriate for immune-prophylaxis or -therapy of several infectious or inflammatory diseases. In line with this, in some inflammation models, SOCS3 over-expression mitigates inflammatory arthritis induced by antigen/IL-1β or collagen, as well as acute inflammation induced by staphylococcal enterotoxin B and LPS (90, 97, 162). On the other hand, when STAT3 plays a protective role for tissue injury, such as in ConA-induced hepatitis, deletion of SOCS3 is anti-inflammatory. As described above, SOCS3 deficiency in macrophages protects mice from LPS-shock because of the enhanced anti-inflammatory effect of STAT3 (80). The down-modulation of SOCS3 expression in CD4+ T cells might be effective in preventing the development of allergic asthma (76).

Therapeutic trials using SOCS3-specific anti-sense oligonucleotides, small hairpin RNAs, or cell-penetrating SOCS3 proteins, have been performed (97, 132, 163). However, to our knowledge the use of small molecules to specifically target SOCS3 have not been reported.

On the other hand, several inhibitors of STAT3, modulating either upstream positive or negative regulators, regulating RNA (DN-STAT3, anti-sense RNA, siRNA) and micro RNA, or small molecules targeting STAT3 at different domains have been approached (164, 165) principally to target constitutive STAT3 activation, which is associated with various human cancers and commonly suggests poor prognosis (166), although attempts to use it in infections or inflammation have not been done. Such inhibitors could also be used in some of the inflammatory or infectious diseases described above to regulate SOCS3 effects.

## Conflict of Interest Statement

The authors declare that the research was conducted in the absence of any commercial or financial relationships that could be construed as a potential conflict of interest.
